# Ivy Leaf Dry Extract EA 575^®^ Has an Inhibitory Effect on the Signalling Cascade of Adenosine Receptor A_2B_

**DOI:** 10.3390/ijms241512373

**Published:** 2023-08-03

**Authors:** Fabio Meurer, Hanns Häberlein, Sebastian Franken

**Affiliations:** Institute of Biochemistry and Molecular Biology, Medical Faculty, University of Bonn, 53115 Bonn, Germany; fameur@uni-bonn.de (F.M.); haeberlein@uni-bonn.de (H.H.)

**Keywords:** ivy leaf dry extract, EA 575^®^, adenosine receptor A_2B_

## Abstract

Ivy leaf dry extract EA 575^®^ is used to improve complaints of chronic inflammatory bronchial diseases and acute inflammation of the respiratory tract accompanied by coughing. Its mechanism of action has so far been explained by influencing β_2_-adrenergic signal transduction. In the present study, we investigated a possible influence on adenosine receptor A_2B_ (A_2B_AR) signalling, as it has been described to play a significant and detrimental role in chronic inflammatory airway diseases. The influence of EA 575^®^ on A_2B_AR signalling was assessed with measurements of dynamic mass redistribution. Subsequently, the effects on A_2B_AR-mediated second messenger cAMP levels, β-arrestin 2 recruitment, and cAMP response element (CRE) activation were examined using luciferase-based HEK293 reporter cell lines. Lastly, the impact on A_2B_AR-mediated IL-6 release in Calu-3 epithelial lung cells was investigated via the Lumit™ Immunoassay. Additionally, the adenosine receptor subtype mediating these effects was specified, and A_2B_AR was found to be responsible. The present study demonstrates an inhibitory influence of EA 575^®^ on A_2B_AR-mediated general cellular response, cAMP levels, β-arrestin 2 recruitment, CRE activation, and IL-6 release. Since these EA 575^®^-mediated effects occur within a time frame of several hours of incubation, its mode of action can be described as indirect. The present data are the first to describe an inhibitory effect of EA 575^®^ on A_2B_AR signalling. This may offer an explanation for the beneficial clinical effects of the extract in adjuvant asthma therapy.

## 1. Introduction

The use of medicinal products containing ivy leaf dry extract EA 575^®^ is recommended for the treatment of chronic inflammatory airway diseases and acute respiratory tract inflammation accompanied by coughing [1,2,3,4]. Until now, the main mechanism of action has been explained by influencing the β_2_-adrenergic receptor (β_2_-AR). It has been shown that α-hederin identified in EA 575^®^ indirectly inhibits the GRK2-mediated phosphorylation of β_2_-AR [5], which is the reason for the decrease in the recruitment of β-arrestin 2 by EA 575^®^ [6]. This leads to the inhibition of β_2_-AR internalisation [7,8,9], which, in turn, results in increased β_2_-adrenergic responsiveness, as evidenced by a corresponding increase in receptor binding and cAMP formation [6,8]. This biased signalling by EA 575^®^ provides an explanation for the bronchospasmolytic and secretolytic effects and a reduction in β-arrestin-mediated negative adverse effects [6]. The anti-inflammatory effects of EA 575^®^ are substantiated by the β-arrestin-independent inhibition of NFκB, presumably by the inhibition of IĸBα phosphorylation, leading to decreased IL-6 release [6,10,11].

Since additional receptor classes are involved in the pathogenesis of these respiratory diseases, we investigated other signalling pathways that may be affected by EA 575^®^ and might explain its beneficial effects in the treatment of inflammatory airway diseases. Many authors have described the harmful influence of adenosine via its adenosine receptor A_2B_ (A_2B_AR) in chronic inflammatory airway diseases [12,13,14,15] based on the following findings.

Elevated adenosine concentrations were found in the bronchoalveolar lavage fluid (BALF) and exhaled breath condensate (EBC) of patients with chronic respiratory diseases such as asthma and chronic obstructive pulmonary disease (COPD), indicating increased adenosine levels in the lungs [16,17,18,19]. These elevations of adenosine were correlated to decreased forced expiratory volumes in 1 s (FEV_1_) and higher Global Initiative for Chronic Obstructive Lung Disease (GOLD) stages [19,20]. Inhalation of adenosine or adenosine monophosphate (AMP) causes bronchoconstriction in patients with asthma and COPD but not in healthy subjects [21,22,23]. Remarkably, elevated transcript levels of A_2B_AR were recovered from the lung tissues of patients with severe COPD, pulmonary fibrosis, and pulmonary arterial hypertension (PAH) [24,25]. Further elevation of transcript and A_2B_AR protein levels was determined in the lung tissues of patients with COPD when accompanied by pulmonary hypertension, indicating a correlation with disease severity [26]. 

Several experiments in mouse models provide additional evidence for the detrimental impact of adenosine and A_2B_AR on airway diseases such as asthma, COPD, and pulmonary fibrosis. Genetically altered adenosine deaminase (ADA)-deficient mice developed severe pulmonary inflammation and airway remodelling as observed in these disorders [27,28]. Lowering adenosine concentrations via ADA enzyme therapy ameliorates lung injury, indicating a correlation between adenosine levels and inflammation as well as fibrosis in the lung [29,30]. Attenuation of pulmonary inflammation, fibrosis, and airway enlargement can also be achieved by administration of specific A_2B_AR antagonists [26,31]. Transcript levels of A_2B_AR were also increased in this model and could be decreased by specific antagonism of A_2B_AR [28,31,32].

Similar observations were made in another mouse model. Mice treated with bleomycin exhibited elevated levels of adenosine and transcripts of A_2B_AR in their lungs and developed pulmonary fibrosis and inflammation. This disease progression could be counteracted with specific A_2B_AR antagonists [31,33]. 

The proinflammatory and profibrotic cytokine interleukin-6 (IL-6) is associated with the signalling of adenosine via A_2B_AR and also plays a pivotal role in these pulmonary disorders [13,34]. Patients with asthma exhibit elevated levels of IL-6 in their sputum and BALF compared to healthy subjects [35,36,37] and compared to asymptomatic asthmatics [38,39], who already have higher serum IL-6 concentrations [40]. A further IL-6 increase in serum and BALF occur after allergen inhalation and during allergic attacks [40,41]. Most remarkably, the sputum IL-6 levels of asthmatics inversely correlate with FEV_1_ and peak expiratory flow [37,42,43]. 

Elevations of IL-6 have also been found in the sputum and serum of patients with COPD compared to healthy controls [44,45], further increasing during exacerbations [46]. As in asthmatics, higher IL-6 levels inversely correlate with lung function [45,46]. Furthermore, correlations with disease severity [47], GOLD stage and BODE index [44], and even mortality [48] have been described.

Correspondingly, genetic IL-6-knockout or anti-IL-6 antibody treatment led to reduced pulmonary inflammation and fibrosis in both of the aforementioned mouse models [49,50]. Further evidence for the deleterious interaction of IL-6 and A_2B_AR has been provided by experiments using genetic A_2B_AR-knockouts. While the adenosine receptor agonist NECA caused increased IL-6 secretion in mouse macrophages, reduced IL-6 levels were found in A_2B_AR-knockout mice [51,52]. Bleomycin-treated mice also exhibited elevated IL-6 levels, which could be decreased by A_2B_AR-knockout, resulting in improved lung function as well as attenuated pulmonary fibrosis and hypertension [25,33,53]. Moreover, ADA enzyme therapy in ADA-deficient mice was able to reduce IL-6 levels [50].

The present study demonstrates the influence of the ivy leaf dry extract EA 575^®^ on adenosine receptor A_2B_ signalling and subsequent IL-6 release. These processes have been described to play a significant and detrimental role in chronic inflammatory pulmonary diseases. The findings may provide an explanation for the positive clinical effects of EA 575^®^ in adjuvant asthma therapy by inhibiting A_2B_AR signalling.

## 2. Results

### 2.1. Dynamic Mass Redistribution Measurements

**Figure 1:**
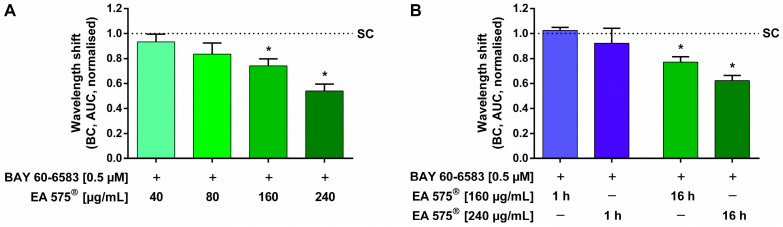
Influence of EA 575^®^ (**A**) and different incubation periods thereof (**B**) on the cellular reaction of HEK cells evoked by stimulation of A_2B_AR. Pre-incubation with different concentrations of EA 575^®^ was performed for 16 h (**A**) and 1 or 16 h (**B**) before cells were stimulated with 0.5 µM BAY 60-6583. The positive wavelength shift mediated by the dynamic mass redistribution of intracellular particles was significantly and dose-dependently inhibited by pre-incubation with 160–240 µg/mL EA 575^®^, compared to stimulated control cells not pre-incubated with EA 575^®^ (SC) (**A**). With an incubation time of 1 h, no reduction in BAY 60-6583-mediated wavelength shift was measured. A significant inhibition was only achieved by pre-incubation with 240 µg/mL EA 575^®^ for 16 h (**B**). Data are shown as baseline-corrected (BC) AUC normalised to stimulated control cells not pre-incubated with EA 575^®^ (SC). Results represent the mean and SEM ((**A**): *n* = 5 independent experiments performed in triplicate; (**B**): *n* = 1 experiment performed in triplicate, * *p* < 0.05).

To investigate the influence of the ivy leaf dry extract, EA 575^®^, on the cellular reaction evoked by the stimulation of A_2B_AR, dynamic mass redistribution (DMR) assays were performed. The cellular reaction of HEK cells to 0.5 µM of the A_2B_AR agonist BAY 60-6583 resulted in a positive wavelength shift mediated by the dynamic mass redistribution of intracellular particles, which was significantly and dose-dependently inhibited up to 45.95 ± 12.36% by pre-incubation with 160–240 µg/mL EA 575^®^ for 16 h (Figure 1A).

In order to examine whether the observed effect results from direct inhibition of the receptor, pre-incubation of HEK cells with EA 575^®^ was conducted for 1 and 16 h. A reduction in wavelength shifts was exclusively observed with 16 h of pre-incubation time, while a shorter incubation period of 1 h did not alter BAY 60-6583-mediated wavelength shifts (Figure 1B).

### 2.2. cAMP Measurements

**Figure 2:**
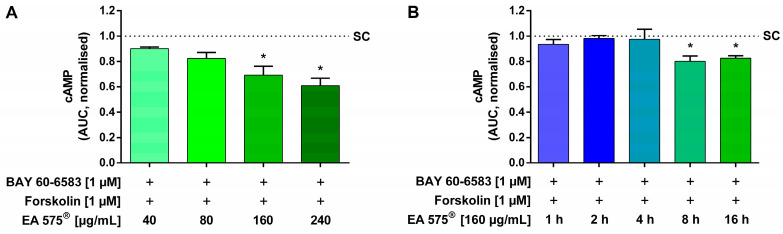
Influence of EA 575^®^ (**A**) and different incubation periods thereof (**B**) on the intracellular cAMP level in HEK GloSensor™ cells elicited by stimulation of A_2B_AR. Prior to co-stimulation with 1 µM BAY 60-6583 and 1 µM forskolin, pre-incubation with different concentrations of EA 575^®^ was performed for 16 h (**A**) and for 1–16 h with 160 µg/mL EA 575^®^ (**B**). The A_2B_AR-induced cAMP increase was significantly and dose-dependently inhibited by pre-incubation with 160–240 µg/mL EA 575^®^, compared to stimulated control cells not pre-incubated with EA 575^®^ (SC) (**A**). Pre-incubation for at least 8 h was necessary to cause a significant decrease in cAMP levels (**B**). Data are presented as AUC normalised to stimulated control cells not pre-incubated with EA 575^®^ (SC). Results represent the mean and SEM (*n* = 3 independent experiments performed in triplicate, * *p* < 0.05).

The influence of EA 575^®^ on the intracellular cAMP level induced by the stimulation of A_2B_AR was determined using HEK cells stably expressing luciferase fused with a cAMP binding domain (GloSensor™, Promega, Mannheim, Germany). Elevation of the second messenger cAMP was elicited by simultaneous stimulation with 1 µM BAY 60-6583 and 1 µM forskolin for one hour. Pre-incubation with 160–240 µg/mL EA 575^®^ for 16 h led to a significant and dose-dependent reduction in cAMP levels by up to 39.02 ± 9.92%, compared to stimulated control cells not pre-incubated with EA 575^®^ (Figure 2A).

In order to determine if the observed effect was mediated by direct inhibition of the receptor and after what time it occurred, different pre-incubation periods were tested. A pre-incubation time of at least 8 h was required to observe a significant decrease in cAMP levels (Figure 2B).

### 2.3. Measurements of β-Arrestin 2 Recruitment

**Figure 3:**
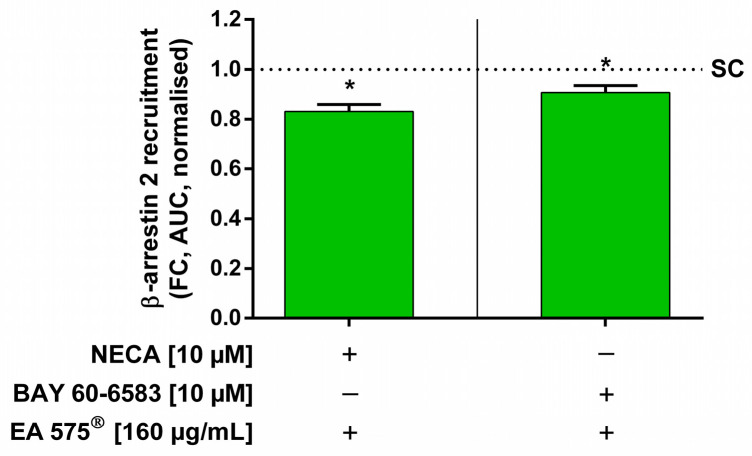
Influence of EA 575^®^ on the recruitment of β-arrestin 2 to the A_2B_AR in transiently transfected HEK cells induced by stimulation with NECA or BAY 60-6583. Pre-incubation with 160 µg/mL EA 575^®^ was conducted for 16 h before cells were stimulated with 10 µM NECA or 10 µM BAY 60-6583. EA 575^®^ was able to significantly inhibit the recruitment of β-arrestin 2 to the A_2B_AR induced by both NECA and BAY 60-6583. Data are shown as AUC of the fold change (FC) after stimulation normalised to equally stimulated control cells not pre-incubated with EA 575^®^ (SC). Results represent the mean and SEM (*n* = 3 independent experiments performed in triplicate, * *p* < 0.05).

To investigate another possible process involved in the inhibition of A_2B_AR by EA 575^®^, measurements of β-arrestin 2 recruitment were performed. HEK cells transiently expressing A_2B_AR-LgBiT and SmBiT-β-arrestin 2 generated a luminescence signal after stimulation with the unspecific adenosine receptor agonist NECA or BAY 60-6583, indicating β-arrestin 2 recruitment to A_2B_AR. Pre-incubation with 160 µg/mL EA 575^®^ for 16 h led to significant inhibition of both NECA- and BAY 60-6583-mediated β-arrestin 2 recruitment by 16.90 ± 4.69% and 9.31 ± 4.82%, respectively (Figure 3).

### 2.4. Measurements of CRE Activation

The influence of EA 575^®^ on cAMP response elements (CRE) was investigated using HEK cells transiently expressing NanoLuc^®^-PEST under the control of a promoter with cAMP response elements.

**Figure 4:**
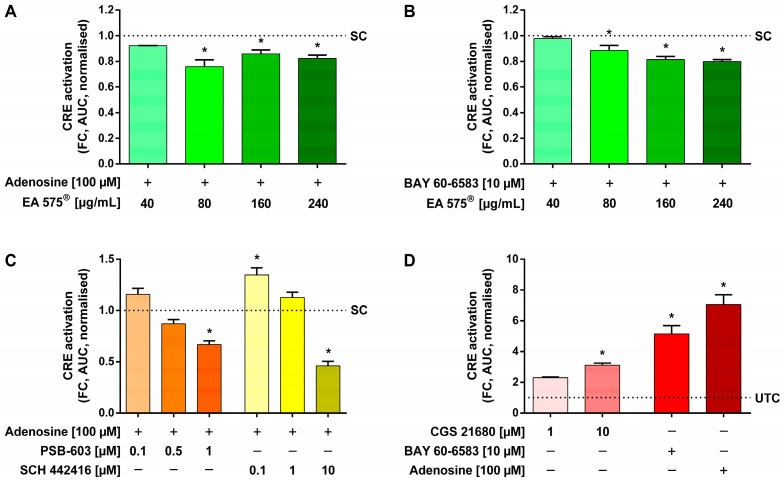
Influence of EA 575^®^ (**A**,**B**) or the antagonists PSB-603 and SCH 442416 (**C**) on the CRE activation in transiently transfected HEK cells mediated by stimulation with adenosine (**A**,**C**) or BAY 60-6583 (**B**). Pre-incubation with 40–240 µg/mL EA 575^®^ was conducted for 16 h (**A**,**B**) or with 0.1–10 µM of the antagonists for 2 h (**C**) before cells were stimulated by adding 100 µM adenosine (**A**,**C**) or 10 µM BAY 60-6583 (**B**). The non-specifically mediated CRE activation was significantly inhibited by pre-incubation with 80–240 µg/mL EA 575^®^ (**A**) or 1 µM PSB-603 (**C**), compared to stimulated control cells not pre-incubated with EA 575^®^ (SC). SCH 442416 also significantly reduced the CRE activation at a concentration of 10 µM, whereas this effect could not be observed at lower concentrations of both antagonists. Instead, 0.1 µM SCH 442416 slightly increased the CRE activation (**C**). The inhibition of the specific A_2B_AR-mediated CRE activation was achieved by pre-incubation with 80–240 µg/mL EA 575^®^ (**B**). Influence of the A_2A_AR agonist CGS 21680 and, in comparison, BAY 60-6583 and adenosine on the CRE activation in transiently transfected HEK cells (**D**). Stimulation was performed with 1–10 µM CGS 21680, 10 µM BAY 60-6583, or 100 µM adenosine. The A_2A_AR-mediated CRE activation was significantly increased by 10 µM CGS 21680 compared with completely untreated control cells (UTC), but to a lesser extent than that mediated by A_2B_AR or non-specifically using adenosine (**D**). Data are shown as AUC of the fold change (FC) after stimulation normalised to stimulated control cells not pre-incubated with EA 575^®^ (SC) (**A**–**C**) or completely untreated control cells (UTC) (**D**). Results represent the mean and SEM (*n* = 3 independent experiments performed in triplicate, * *p* < 0.05).

The impact of EA 575^®^ on adenosine-mediated CRE activation was examined first. Compared to stimulated control cells not pre-incubated with EA 575^®^, pre-incubation with 80–240 µg/mL EA 575^®^ for 16 h led to a significant reduction by a maximum of 24.23 ± 9.38% of the CRE activation mediated by stimulation with 100 µM adenosine (Figure 4A). Pre-incubation with EA 575^®^ also specifically inhibited A_2B_AR-mediated CRE activation by 10 µM BAY 60-6583 in a dose-dependent manner up to 20.18 ± 2.67%, being significant at 80–240 µg/mL (Figure 4B).

Since adenosine is a non-specific agonist binding to all adenosine receptor subtypes, identification of the receptor accountable for the observed effect was addressed next. For this purpose, cells were pre-incubated with the A_2B_AR-specific antagonist PSB-603 or the A_2A_AR-specific antagonist SCH 442416 for one hour prior to stimulation with 100 µM adenosine. PSB-603 was able to significantly inhibit the adenosine-mediated CRE activation by 33.37 ± 6.63% at a concentration of 1 µM, while SCH 442416 significantly reduced the CRE activation by 54.11 ± 7.90% at a concentration of 10 µM. Lower concentrations of both antagonists did not show a significant reduction in the signal, whereas 0.1 µM SCH 442416 significantly increased CRE activation by 34.64 ± 12.25% (Figure 4C).

CRE activation could also be mediated by stimulation with 10 µM of the A_2A_AR agonist CGS 21680, resulting in a significant 3.10 ± 0.24-fold increase in the luminescence signal compared to completely untreated control cells. Nevertheless, this A_2A_AR stimulation elicited a smaller effect than 10 µM BAY 60-6583, which showed a 5.14 ± 0.93-fold elevation, and 100 µM adenosine, for which the signal was highest with a 7.06 ± 1.09-fold increase (Figure 4D).

### 2.5. IL-6 Measurements

IL-6 release of Calu-3 cells was provoked by stimulation with 100 µM adenosine and measured as luminescence using the Lumit™ IL-6 (Human) Immunoassay by Promega.

**Figure 5:**
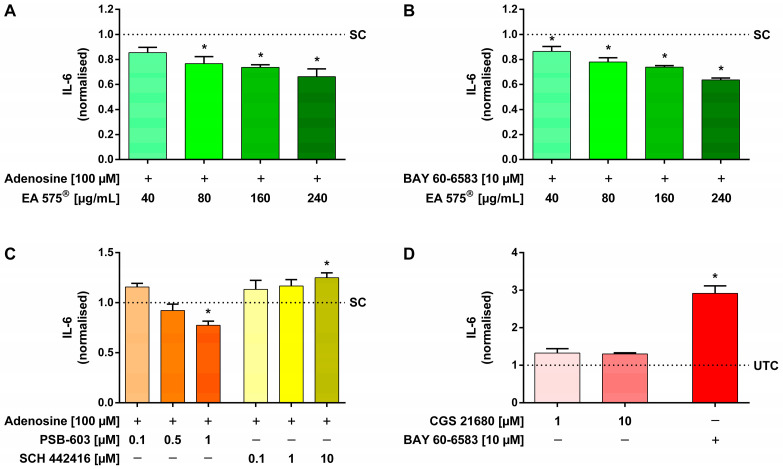
Influence of EA 575^®^ (**A**,**B**) or the antagonists PSB-603 and SCH 442416 (**C**) on the IL-6 release of Calu-3 cells mediated by stimulation with adenosine (**A**,**C**) or BAY 60-6583 (**B**). Pre-incubation with 40–240 µg/mL EA 575^®^ was conducted for 16 h (**A**,**B**) or with 0.1–10 µM of the antagonists for 1 h (**C**) before cells were stimulated by adding 100 µM adenosine (**A**,**C**) or 10 µM BAY 60-6583 (**B**) for another 24 h. The non-specifically mediated IL-6 release was significantly and dose-dependently inhibited by pre-incubation with 80–240 µg/mL EA 575^®^ (A) or 1 µM PSB-603 (**C**), compared to stimulated control cells not pre-incubated with EA 575^®^ (SC). SCH 442416, however, increased the concentration of IL-6 even further. (**C**). The inhibition of the specific A_2B_AR-mediated IL-6 release was achieved by pre-incubation with 40–240 µg/mL EA 575^®^ (**B**). Influence of the A_2A_AR agonist CGS 21680 and, in comparison, BAY 60-6583 on the IL-6 release of Calu-3 cells (**D**). Stimulation was performed with 1–10 µM CGS 21680 or 10 µM BAY 60-6583 for 24 h. The A_2A_AR-mediated IL-6 release was slightly, but neither significantly nor dose-dependently, increased compared to completely untreated control cells (UTC) (**D**). Results represent the mean normalised to stimulated control cells not pre-incubated with EA 575^®^ (SC) (**A**–**C**) or completely untreated control cells (UTC) (**D**) and SEM (*n* = 3 independent experiments performed in triplicate, * *p* < 0.05).

The influence of EA 575^®^ on this non-specifically mediated IL-6 release was investigated. Compared to stimulated control cells not pre-incubated with EA 575^®^, pre-incubation with 80–240 µg/mL EA 575^®^ for 16 h led to a significant and dose-dependent reduction by up to 33.73 ± 10.76% of the adenosine-mediated IL-6 release (Figure 5A). Also, when pre-incubating with 40–240 µg/mL EA 575^®^, BAY 60-6583-mediated IL-6 release was significantly inhibited up to 36.36 ± 2.16% in a dose-dependent manner (Figure 5B).

Receptor-specific antagonists PSB-603 and SCH 442416 were employed to identify the specific receptor subtype involved in the induction of adenosine-mediated IL-6 release. PSB-603 was able to significantly inhibit the adenosine-mediated IL-6 release by 22.56 ± 8.19% at a concentration of 1 µM, whereas SCH 442416 increased the concentration of IL-6 even further by up to 24.99 ± 9.57% (Figure 5C).

In another approach, stimulation was performed with 10 µM BAY 60-6583, resulting in a 2.91 ± 0.35-fold increase in IL-6 release, while A_2A_AR agonist CGS 21680 had no significant effect, showing a maximal 1.33 ± 0.20-fold IL-6 increase, compared to completely untreated control cells (Figure 5D).

### 2.6. Measurements of NFκB Transcriptional Activity

**Figure 6:**
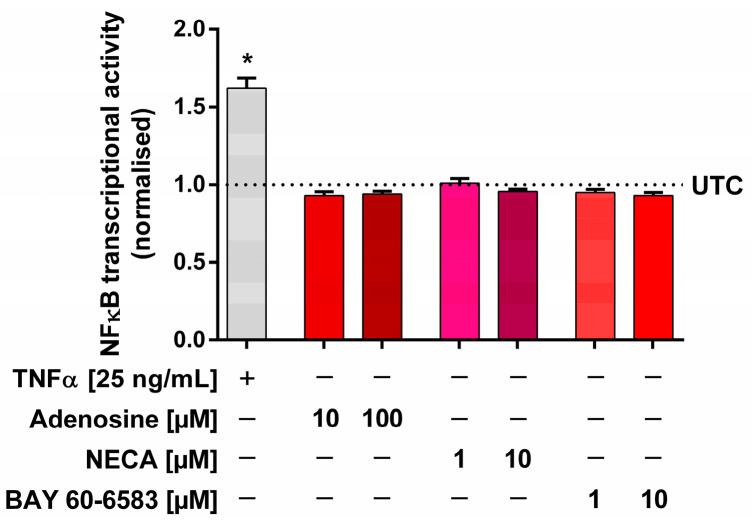
Influence of different adenosine receptor agonists on NFκB transcriptional activity. Calu-3 cells stably expressing a secreted NanoLuc^®^ under control of an NFκB binding sequence were stimulated for 3 h. An amount of 25 ng/mL TNFα was used as positive control, resulting in a significant increase in the luminescence signal. Amounts of 10–100 µM adenosine, 1–10 µM NECA, or 1–10 µM BAY 60-6583 did not show any effect on the NFκB transcriptional activity. Results represent the mean normalised to completely untreated control cells (UTC) and SEM (*n* = 3 independent experiments performed in triplicate, * *p* < 0.05).

In order to evaluate whether the observed effect of an IL-6 release via adenosine receptors in Calu-3 cells was NFκB-dependent, the influence of adenosine receptor agonists on NFκB transcriptional activity was investigated using an NFκB reporter gene cell line. Calu-3 cells stably expressing a secreted NanoLuc^®^ under the control of an NFκB-binding sequence reacted with a 1.62 ± 0.11-fold increase in the luminescence signal to stimulation with 25 ng/mL TNFα for 3 h. However, neither 10–100 µM adenosine, nor 1–10 µM NECA, nor 1–10 µM BAY 60-6583 showed any effect on the NFκB transcriptional activity (Figure 6).

## 3. Discussion

In the present study, we further explored the mechanism of action of the ivy leaf dry extract EA 575^®^ to explain its positive effects on inflammatory airway diseases [2,3,4]. Since adenosine receptor A_2B_ (A_2B_AR) plays an essential role in the pathogenesis of chronic inflammatory airway diseases such as asthma, COPD, and pulmonary fibrosis [12,13,14,15], we investigated a possible effect of EA 575^®^ on this receptor.

We first examined a possible impact of EA 575^®^ on the cellular response of HEK cells to stimulation of the A_2B_AR using DMR measurements. This label-free technology is well-suited for an initial investigation of the general influence of ligands on GPCR signalling due to its capability to provide a holistic overview of the complex cellular response [54]. We observed the dose-dependent inhibition of the cellular reaction to A_2B_AR stimulation by EA 575^®^. This effect was observed after 16 h of pre-incubation, while no impact was seen after a short incubation time of 1 h, indicating indirect inhibition of A_2B_AR.

To further specify the influenced cellular response observed in the DMR experiments, second messenger cAMP was assessed. In alignment with the DMR results, a corresponding decrease in cAMP levels was observed after pre-treatment with EA 575^®^ under A_2B_AR stimulatory conditions. A similar effect was shown for the highly selective A_2B_AR antagonist PSB-603 using the same cAMP biosensor technology [55]. Next, we challenged the required minimum incubation time period and found that at least 8 h of EA 575^®^ pre-incubation was necessary to mediate a reduction in cAMP levels. Such long incubation periods are unlikely to be in accordance with a direct mode of action, as these ligands typically compete for receptor binding sites within minutes. PSB-603, for example, requires a pre-incubation period of only 30 min in this assay [55]. These findings confirm that EA 575^®^ is able to inhibit the A_2B_AR signalling pathway and that this can be explained by an indirect mechanism of action. Since adenosine receptors are ubiquitously expressed in humans, this could be advantageous over orthosteric antagonism in terms of adverse effects [56]. To our knowledge, the present study is the first to show the influence of an ivy extract on adenosine receptor A_2B_.

To further investigate the mode of action of EA 575^®^ on A_2B_AR and to determine whether it also influences other downstream signalling pathways, the recruitment of β-arrestin 2 to A_2B_AR was examined. For this purpose, a slightly modified assay system recently described by Saecker et al. was used [57]. In this system, A_2B_AR is tagged with LgBiT and β-arrestin 2 with SmBiT. When β-arrestin 2 recruitment occurs specifically to A_2B_AR, luminescence is generated by reversible complementation of a nanoluciferase. Stimulation was performed using the unspecific adenosine receptor agonist NECA, as well as the specific A_2B_AR agonist BAY 60-6583. Since A_2B_AR is the only receptor tagged with LgBiT in this system, the effects detected after stimulation with NECA can also be considered specific for this receptor in this case. EA 575^®^ caused a reduction in β-arrestin 2 recruitment to A_2B_AR and thus affected the receptor signalling in an inhibitory manner in several ways. This is remarkable as under β_2_-AR stimulatory conditions, EA 575^®^ inhibits β-arrestin 2 recruitment while enhancing G protein/cAMP signalling [6].

In the downstream signalling cascade of G_s_ protein-coupled receptors such as A_2B_AR, cAMP causes phosphorylation of cAMP response element-binding protein via PKA, PKC, and ERK, and can thereby activate cAMP response elements (CRE) located in promoter regions, thus affecting the transcriptional activity of genes [58,59,60,61]. Since EA 575^®^ reduced cAMP levels under A_2B_AR stimulatory conditions, subsequent inhibition of CRE was expected. Indeed, EA 575^®^ inhibited CRE activation mediated both non-specifically by adenosine as well as A_2B_AR-specifically by BAY 60-6583. A similar result was found for a specific A_2B_AR antagonist, which reduced the NECA-mediated phosphorylation of CRE binding protein [62].

One of the genes regulated by CRE, encoding IL-6, plays a crucial role in several inflammatory and airway diseases, such as asthma and COPD [34]. The IL-6 promoter region contains several elements that activate IL-6 expression, one of which is a CRE [63,64,65,66,67]. The release of IL-6 and other inflammatory and chemotactic mediators, in turn, can be mediated by adenosine via the A_2B_AR signalling pathway, and CRE might be at least one important factor in this signalling cascade [68,69]. Since EA 575^®^ inhibits both A_2B_AR signalling and CRE activation, an effect on IL-6 release seemed plausible. Therefore, we tested the potential effect of EA 575^®^ on the adenosine-mediated IL-6 release in Calu-3 cells. It was found that EA 575^®^, in fact, reduces the adenosine-mediated release of IL-6, indicating a possible reduction in IL-6-mediated airway inflammation and fibrosis. This inhibition of A_2B_AR-mediated IL-6 release could also be reproduced with the specific agonist BAY 60-6583.

This is a new finding that complements the previously published decrease in IL-6 release by EA 575^®^ via inhibition of NFκB. In our experiments, we were able to demonstrate that neither adenosine, NECA, nor BAY 60-6583 influenced NFκB transcriptional activity in Calu-3 cells. Although the IL-6 promoter region contains an NFκB binding element [70,71], the release of IL-6 via adenosine receptors is not mediated by this promoter element. This is in line with the results published by Sitaraman et al., who showed that the NFκB binding site, in contrast to the CRE binding site, is not important for adenosine-mediated IL-6 release [69]. Similarly, Zhong et al. found that NECA does not affect NFκB-mediated transcription but rather affects CRE-mediated transcription [68]. Therefore, in this case, EA 575^®^ affects IL-6 release via another mechanism, which could be the inhibition of CRE.

Additionally, we wanted to specify which adenosine receptor subtype is responsible for the observed effects in the IL-6 and CRE activation assays. Therefore, we investigated the inhibition of the adenosine-mediated CRE activation and IL-6 release with the A_2A_AR antagonist SCH 442416 or the A_2B_AR antagonist PSB-603, as these are the predominantly expressed adenosine receptor subtypes in the cell types we used [72,73,74]. Only PSB-603 reduced both CRE activation and IL-6 release after stimulation with adenosine. In contrast, SCH 442416 caused the inhibition of CRE activation but simultaneously led to a slight increase in IL-6 release. In addition, CGS 21680, a specific A_2A_AR agonist, was shown to increase CRE activation in a dose-dependent manner but had no effect on IL-6 release. These data suggest that A_2A_AR signalling activates CRE but does not result in an increase in IL-6. This might be because IL-6 is regulated not only by the cAMP response element but also by several other elements, as mentioned above. These findings suggest that adenosine mediates IL-6 release through CRE activation via A_2B_AR. Furthermore, adenosine mediates IL-6 release only in concentrations as high as 10–100 µM [61,68,69,75]. Considering the affinities of adenosine to the different receptor subtypes (EC_50_: A_1_ = 0.31 µM, A_2A_ = 0.73 µM, A_2B_ = 23.5 µM, A_3_ = 0.29 µM) [76], this also suggests mediation via A_2B_AR. Taken together, published receptor-affinity data and our results indicate that adenosine-induced IL-6 release is mediated through the A_2B_AR signalling pathway. These findings disagree with those of Sun et al., who stated that A_2A_AR, but not A_2B_AR, is responsible for adenosine-mediated IL-6 release [61], but are consistent with data from Sitaraman et al. and Zhong et al., who both identified A_2B_AR as being responsible for adenosine-mediated IL-6 release [68,69,75]. Although not particularly distinguishing between the two receptors, several other studies have provided additional evidence for the A_2B_AR-mediated inhibition of IL-6 release. Elevated IL-6 levels in ADA-deficient and bleomycin-treated mice were reduced by the administration of a specific A_2B_AR antagonist [31,33]. Additionally, a NECA-mediated increase in IL-6 was reduced by antagonists of A_2B_AR in these models [33,50]. A reduction in NECA-mediated IL-6 elevation by A_2B_AR antagonists was also shown in macrophages [24,52]. Secretion of IL-6 after treating pulmonary arterial smooth muscle cells of PAH patients with BAY 60-6583 under hypoxic conditions was also reduced by a specific antagonist of A_2B_AR [25]. 

Moreover, A_2A_AR signalling is described as anti-inflammatory and lung protective, which basically matches our results, suggesting that this receptor does not contribute to IL-6 release but rather attenuates it [12,13,14]. Nevertheless, our findings differ from others in terms of the influence of A_2A_AR on IL-6. There have been reports of both increases [77] as well as reductions [78,79,80,81] by the stimulation of A_2A_AR with CGS 21680, whereas other research, similar to our study, found no effect [82,83]. Furthermore, the genetic knockout of A_2A_AR in mice led to higher expressions of IL-6 in one study [84] but did not affect IL-6 in another study examining an ADA/A_2A_AR double knockout [85]. However, in other studies, antagonism of A_2A_AR caused the elevation of IL-6 levels, matching our findings [79,80,82].

Several factors may account for these controversial findings. First, the effect of adenosine may be dependent on its concentration and the stage of the disease. At low levels, adenosine activates high-affinity receptors such as A_2A_AR, triggering anti-inflammatory pathways. In highly inflammatory environments and chronic disease states such as asthma or COPD, higher levels of adenosine are released. Adenosine concentrations have been estimated to reach about 200 µM in the lungs of asthmatics [16]. At such high concentrations, low-affinity A_2B_AR is activated, which may lead to further exacerbation of airway inflammation [15]. Inhibition of A_2B_AR is therefore considered beneficial in chronic inflammatory airway diseases [12,13,14]. In a guinea pig model of asthma, antagonism of A_2B_AR ameliorated the changes provoked by an allergen challenge, whereas A_2A_AR antagonism deteriorated them [86]. A_2B_AR antagonism also proved beneficial in mouse models of chronic lung diseases as it attenuated bronchoconstriction, airway inflammation, pulmonary fibrosis, and airspace enlargement [26,31,33,87].

In conclusion, this offers a possible explanation for the positive clinical effects of the extract in adjuvant asthma therapy by means of a possible reduction in adenosine-mediated inflammation and bronchoconstriction [88]. Still, further research is necessary to fully understand the mechanisms of action of this versatile extract. It is still unclear which constituents play a role in the observed effects. It has been shown that a fraction of an ivy leaf extract enriched in phenolics and flavonoids elicits anti-inflammatory properties [89,90]. Since some of the enriched substances have also been identified in EA 575^®^, future investigations should be performed with the pure compounds. However, the results presented here provide a rationale for further studies in animals or human subjects to prove further effects regarding chronic airway diseases such as asthma or COPD.

## 4. Materials and Methods

### 4.1. Chemicals

Ivy leaf dry extract EA 575^®^ (DER 5-7.5:1, 30% ethanol; batch number 14B0310) was received from Engelhard Arzneimittel (Niederdorfelden, Germany) and is well characterised by 17 ingredients from the natural product classes of flavonoids, saponins, and dicaffeoylquinic acids identified via LC-MS analysis [7]. All reagents for luciferase assays were received from Promega (Mannheim, Germany) if not stated otherwise. Coelenterazine h was obtained from Prolume (Pinetop-Lakeside, AZ, USA). Adenosine, BAY 60-6583, forskolin, 5′-N-Ethylcarboxamidoadenosine (NECA), and SCH 442416 were obtained from Sigma-Aldrich (Crailsheim, Germany). TNFα was received from Merck (Darmstadt, Germany). PSB-603 and CGS 21680 were obtained from Biomol (Hamburg, Germany).

### 4.2. Cell Culture

Human embryonic kidney cells (HEK293), subsequently called HEK cells, were obtained from DSMZ (No. ACC 305; Braunschweig, Germany). HEK cells and all constructed clones were cultivated at 37 °C with 5% CO_2_ in DMEM supplemented with 100 units/mL penicillin, 100 µg/mL streptomycin, and 10% fetal bovine serum (FBS) (all obtained from Thermo Fisher Scientific, Waltham, MA, USA). Cells were subcultured 1:10 every 3–4 days in 10 cm cell culture dishes.

Calu-3 cells were obtained from ATCC (HTB-55; Manassas, VA, USA). Calu-3 cells and all constructed clones were cultivated at 37 °C with 5% CO_2_ in DMEM/F-12 supplemented with GlutaMAX™, 100 units/mL penicillin, 100 µg/mL streptomycin, and 15% FBS (all obtained from Thermo Fisher Scientific). Cells were subcultured 1:5 every 5–7 days in 10 cm cell culture dishes.

### 4.3. Dynamic Mass Redistribution Measurements

Dynamic mass redistribution (DMR) measurements were performed using the Corning Epic^®^ biosensor. HEK GloSensor™ cells were seeded at a density of 3000 cells per well in a 384-well plate by Corning (#5042; Corning, NY, USA) and allowed to grow for at least 24 h in full growth medium. Pre-incubation was performed with 40, 80, 160, or 240 µg/mL EA 575^®^ for up to 16 h. After pre-incubation, the medium was replaced by HBSS supplemented with 20 mM HEPES and the cells were allowed to equilibrate at 37 °C for one hour. A baseline of 10 measurement points was recorded before stimulation was performed using a CyBi^®^-SELMA semi-automatic pipetting system (Analytik Jena AG, Jena, Germany) at 37 °C. Subsequently, the wavelength shift mediated by the dynamic mass redistribution of intracellular particles was measured for another 70 min.

### 4.4. cAMP Measurements

The establishment of HEK cells expressing a cAMP sensor and the measurement of cAMP were performed as described by Bussmann et al. [8]. Briefly, pre-incubation was carried out with 40, 80, 160, or 240 µg/mL EA 575^®^ for up to 16 h in full growth medium. After pre-incubation, the medium was changed to a substrate solution containing 4% GloSensor™ cAMP reagent stock solution in HEPES-buffered DMEM. Cells were incubated at 37 °C for one hour and subsequently equilibrated at room temperature in the plate reader (Tecan Infinite^®^ 200 PRO, Tecan, Männedorf, Switzerland) for another hour. Stimulation was performed with 1 μM BAY 60-6583 and 1 μM forskolin simultaneously, and cAMP increase was measured as luminescence for one hour after stimulation.

### 4.5. Measurements of β-Arrestin 2 Recruitment

The plasmid coding for human adenosine receptor A_2B_ (A_2B_AR) fused to the N-terminus of Large BiT (LgBiT) was generated by initially removing the region coding for YFP of the vector *pEYFP-N1-A2BR*, which was a gift from Robert Tarran (Addgene plasmid # 37202; http://n2t.net/addgene:37202 (accessed 13 November 2019); RRID:Addgene_37202) [91], using BamHI/NotI. The open reading frame coding for LgBiT was amplified via PCR (forward primer: 5′-GATCGGATCCAAGTGGTAGCGGGGTCTTTACCCTG-3′; reverse primer: 5′-GATCGCGGCCGCTAGCTACCACCGCATCC-3′). The PCR product was cut with BamHI/NotI and inserted into the vector via ligation.

For expression of rat β-arrestin 2 with an N-terminal Small BiT (SmBiT), the coding sequence was taken from *pECFP-N1-rβ-arrestin-2* (a gift from M. Bouvier, Montreal, QC, Canada) by restriction with NheI/SalI. The fragment was introduced into *pcDNA™3.1/Zeo^(+)^* Mammalian Expression Vector (Invitrogen, Waltham, MA, USA) containing the information for the SmBit via NheI and XhoI sites.

HEK cells were co-transfected to transiently express A_2B_AR-LgBiT and SmBiT-β-arrestin 2 using branched polyethylenimine (PEI) (Sigma-Aldrich). For this purpose, cells were seeded in a 6-well plate at a density of 350,000 cells per well and allowed to grow for one day. For each DNA, 1.5 µg was diluted into 200 µL of 150 mM NaCl, then 7.5 µL of a 1 mg/mL PEI solution was added, and the mixture was vortexed immediately for 10 s. After 10 min at RT, the DNA/PEI mixture was added to the cells and incubated for 24 h. The transiently transfected cells were seeded in a 96-well plate at a density of 15,000 cells per well and allowed to grow for one day in full growth medium. Pre-incubation was carried out with 160 µg/mL EA 575^®^ for 16 h.

In general, measurements of recruitment of β-arrestin 2 to A_2B_AR were performed as recently described by Saecker et al. for A_1_AR [57]. Briefly, pre-incubation medium was replaced by a solution of 2.5 µM coelenterazine h in HBSS supplemented with 20 mM HEPES. A baseline of 3 measurement points was recorded before stimulation was performed. Subsequently, luminescence corresponding to the recruitment of β-arrestin 2 was measured for another 27 min using a Spark^®^ plate reader by Tecan.

### 4.6. Measurements of CRE Activation

The plasmid expressing NanoLuc^®^-PEST (NlucP) from a promoter with cAMP response elements (CRE) was received by Promega. HEK cells were transfected to transiently express this construct using PEI, as described above. Transiently transfected HEK cells were seeded in a 96-well plate at a density of 20,000 cells per well and allowed to grow for one day in fully supplemented medium. Pre-incubation was conducted with 40, 80, 160, or 240 µg/mL EA 575^®^ in full growth medium for 16 h. Pre-incubation with antagonists was carried out for only two hours, simultaneously with the substrate incubation. Nano-Glo^®^ Vivazine™ Live Cell Substrate (Promega) was prepared according to the manufacturer’s instructions using HEPES-buffered medium, and cells were incubated with the substrate for 2 h at 37 °C in a Tecan Spark^®^ plate reader already measuring luminescence. Stimulation was then performed by adding adenosine, BAY 60-6583, or CGS 21680, and measurement was performed for another 22 h.

### 4.7. IL-6 Measurements

Measurement of IL-6 release was performed using the Lumit™ IL-6 (Human) Immunoassay by Promega. Calu-3 cells were seeded in a 96-well plate at a density of 20,000 cells per well and allowed to grow for at least two days to a confluency of 80–90% in full growth medium. Pre-incubation was carried out with 40, 80, 160, or 240 µg/mL EA 575^®^ in DMEM/F-12 without phenol red supplemented with GlutaMAX™ and 5% FBS for 16 h. Pre-incubation with antagonists was conducted for only one hour. IL-6 release was then provoked by adding adenosine, BAY 60-6583, or CGS 21680 for the following 24 h. Subsequently, the measurement of IL-6 was performed according to the manufacturer’s instructions using a Tecan Spark^®^ plate reader.

### 4.8. Measurements of NFκB Transcriptional Activity

For the generation of a secreted NanoLuc^®^ expression construct under the control of an NFκB binding sequence, the *pNFkB-d2EGFP* vector (Clontech, Mountain View, CA, USA) was used as a first template. Destabilised GFP was removed from the vector by PCR (forward primer: 5′-TCGGATATCTCGAGCCGGAATTCGGGGAAGCTTC-3′; reverse primer: 5′-GTTCAGGGGGAGGTGTG-3′) and restriction with BamHI/XhoI. The open reading frame coding for the secreted NanoLuc^®^ was cut from *pNL1.3[secNluc]* vector (Promega) using BamHI/XhoI and introduced into the vector via ligation. 

In a second step, this secreted NanoLuc^®^ expression construct, under the control of an NFκB binding sequence, was cloned into the *pcDNA™3.1^(+)^* Mammalian Expression Vector (Invitrogen). Therefore, the CMV promoter was removed from the vector by restriction with BamHI/BglII and re-ligation. Then, the vector was re-cut with NotI/XhoI. The insert was isolated out of the plasmid generated in the first step using NotI/SalI and introduced into the vector via ligation.

Calu-3 cells were transfected using Metafectene^®^ Pro (Biontex, Munich, Germany) according to the manufacturer’s instructions. For the selection of successfully transfected cells, the medium was changed to fully supplemented DMEM/F-12 containing 600 µg/mL G418 (Thermo Fisher Scientific).

Cells were seeded in a 96-well plate at a density of 25,000 cells per well and allowed to grow for at least two days to a confluency of 80–90% in full growth medium. Before stimulating with TNFα, adenosine, NECA, or BAY 60-6583 for 3 h, cells were starved overnight. Measurement of NFκB transcriptional activity was performed in a Tecan Spark^®^ plate reader using the Nano-Glo^®^ Luciferase Assay System (Promega) according to the manufacturer’s instructions.

### 4.9. Statistical Analysis

For statistical analysis, one-way analysis of variance (ANOVA) with Dunnett’s multiple comparisons test was performed using Prism software version 6.01 (GraphPad Software, San Diego, CA, USA). Results were considered to be significant for *p*-values of <0.05.

## Data Availability

All relevant data are contained within the article.
